# Impact of *Candida auris* on Critically Ill Patients: A Three-Year Observational Study in a Greek Intensive Care Unit

**DOI:** 10.3390/pathogens14040328

**Published:** 2025-03-28

**Authors:** Maria Katsiari, Charikleia Nikolaou, Eleftheria Palla, Kalliopi Theodoridou, Athanasios Tsakris, Georgia Vrioni

**Affiliations:** 1 Intensive Care Unit, Konstantopouleio-Patision General Hospital, 3-5 Theodorou Konstantopoulou Street, N. Ionia, 14233 Athens, Greece; hariklia2009@yahoo.gr; 2 Department of Microbiology, Konstantopouleio- Patision General Hospital, 3-5 Theodorou Konstantopoulou Street, N. Ionia, 14233 Athens, Greece; elefthepal@gmail.com; 3 Department of Microbiology, Medical School, National and Kapodistrian University of Athens, 75 Mikras Asias Street, 11527 Athens, Greece; lmktheo@yahoo.com (K.T.); atsakris@med.uoa.gr (A.T.)

**Keywords:** *candida auris*, intensive care unit, risk factors, MALDI-TOF, antifungal resistance, ICU mortality

## Abstract

*Candida auris* has emerged as a multidrug-resistant yeast implicated in healthcare-associated invasive infections and hospital outbreaks. The aim of the current 38-month period observational study in a multidisciplinary Intensive Care Unit (ICU) was to analyze the epidemiology, potential risk factors, management strategies, and patient outcomes of patients with *C. auris*. During the study period, 32 patients were identified with *C. auris* infection (6 patients) or colonization (26 patients) and their clinical characteristics and treatment-related factors were compared. Identification of *C. auris* isolates was confirmed by MALDI-TOF spectrometry. According to our results, regarding patient-related factors, no significant differences were identified. Regarding treatment-related factors, the proportion of patients already receiving corticosteroids (34.6% vs. 83.3%, *p* = 0.064) or being on renal replacement treatment (7.7% vs. 33.3%) was higher in infected patients. Median time elapsed from ICU admission to first positive culture was 7 (1–21) days and half of cases were ICU-imported. All strains were resistant to fluconazole and susceptible to echinocandines and amphotericin B. Crude mortality of the study population was 43.75%, similar to other previously reported candidemias. Rapid identification of *C. auris*, continued surveillance, and infection control practices are important elements for controlling successfully its spread in the hospital setting and for establishing promptly its transition from commensalism to infection.

## 1. Introduction

*Candida auris* is a multidrug-resistant fungal pathogen that has emerged as a global threat in the last decade. Since its first report in 2009, after being isolated from the external ear canal discharge of a patient in Japan [1], it has been implicated in nosocomial outbreaks worldwide, often in Intensive Care Units (ICUs) [2,3]. Based on whole-genome sequencing, six distinct clades were classified by region of independent emergence: Clade I (South Asian), Clade II (East Asian), Clade III (African), Clade IV (South American), Clade V (Iranian), and Clade VI (Bangladesh-Singapore) [4,5]. These clades depict different genetic determinants of resistance and resulting antifungal resistance profiles. Although specific clades of *C. auris* continue to dominate the parts of the world where they originated, transmission in other areas has been reported, for example, in China, where three different genetic clades (I, II, III) have been identified in 18 hospitals during 2018–2023 [6,7,8].

The earliest *C. auris* isolate was uncovered in South Korea in 1996, as a misidentified isolate from a bloodstream infection in a pediatric surgery patient [9], whereas a 2008 isolate from Pakistan was also recognized [10]. A reanalysis of 20,788 *Candida* spp. isolates collected from four continents by the SENTRY Antifungal Surveillance Program between 1997 and 2016, did not find *C. auris* isolates before 2009, and only six misidentified isolates between 2009 and 2016 [11]. These data support the emergence of *C. auris* as a relatively recent clinical problem rather than its misidentification previously, due to a reliance on conventional phenotypic methods.

*C. auris* displays characteristics, such as multidrug resistance, that are evocative of other yeasts (e.g., *Yarrowia lypolitica*, *Stephanoascus ciferrii* complex, and *Candida blankie*) or bacteria, as well as exceptional adaptation to the nosocomial environment and spread between patients and hospitals [12,13,14,15,16]. *C. auris*’ ability to persist on healthcare surfaces and medical equipment, along with its propensity for persistent skin colonization, may account for its high transmissibility [7,17,18]. Consequently, preventing the spread of *C. auris* depends on prompt and accurate identification of cases and the implementation of infection control precautions. Unfortunately, the identification of *C. auris* remains challenging since the most common diagnostic platforms available in clinical and public health laboratories (e.g., VITEK 2) often misidentify *C. auris* [19,20,21,22]. Accurate identification can be accomplished with matrix-assisted laser desorption/ionization time-of-flight (MALDI-TOF) mass spectrometry [19], ribosomal DNA sequencing [4], polymerase chain reaction [23,24], and T2 Magnetic Resonance assay [25].

*C. auris*’ high-level resistance to antifungals complicates the successful management of their corresponding infections. Further concern causes the lack of *C. auris*-specific interpretative breakpoints, yet to be reported by both the Clinical Laboratory Standards Institute (CLSI) and the European Committee for Antimicrobial Susceptibility Testing (EUCAST). However, tentative interpretative criteria have been proposed by the U.S.A. Centers for Disease Control and Prevention (CDC) [26]. Based on these breakpoints, most isolates are resistant to fluconazole (90%) and exhibit variable susceptibility to amphotericin B (30%) and echinocandines (5%). Multidrug resistance has been reported in 41% of the strains and pan-resistance in 3–4% [12,27], with clade-specific variations [6,12].

In Greece, the first report of *C. auris* in 2019 involved a sporadic case of a 20-year-old male cystic fibrosis patient who presented with respiratory exacerbation [28]. Thereafter, the isolation of *C. auris* strains exhibited exponential growth and involved several hospitals within various prefectures. The aim of the current 38-month period observational study in a multidisciplinary ICU was to analyze the epidemiology, potential risk factors, management strategies, and patient outcomes of patients with *C. auris*.

## 2. Materials and Methods

### 2.1. Setting

Konstantopouleio-Patission is a 330-bed tertiary care hospital in Athens, Greece, which includes a 9-bed multidisciplinary ICU, internal medicine, cardiology, surgical, urology, orthopaedical, and other wards. In this retrospective observational study, we studied the patients who were colonized or infected with *C. auris* between 10 November 2020 and 31 December 2023. The ICU was converted to a COVID-19 unit on 25 February 2021, during the third wave of COVID-19 in Greece, and operated in this way for 15 consecutive months.

### 2.2. Definitions of Cases and Collection of Clinical Data

A case was defined as any patient hospitalized in the ICU and colonized or infected with *C. auris* during routine surveillance or targeted cultures from various clinical specimens. Colonization was defined as the presence of *C. auris* in samples obtained from urine, tracheal aspirates, and swab specimens from the skin and rectum. Infection was defined as the growth of *C. auris* in blood culture or a sample obtained from a sterile site, along with compatible clinical signs of infection. Patients underwent screening on admission to the ICU and then on a weekly basis. Screening involved culture swab specimens from the axilla, groin, and rectum, as well as urine and tracheal aspirates cultures. Blood cultures were aspirated based on clinical criteria of sepsis [29]. When multiple specimens of a patient showed growth of *C. auris*, only the specimen which was identified as a source of infection was considered. When *C. auris* was isolated from multiple cultures of a single patient, only the first isolate was included in the study.

Clinical and epidemiological data of ICU patients were reviewed, including gender, age, initial diagnosis, surgical procedures, disease severity at ICU admission as determined by an Acute Physiology Chronic Health Evaluation (APACHE II) score, comorbidities, previous treatment with antibiotics and antifungals, outcome and length of stay (LOS) in the ICU, and outcome on the 28th day after ICU discharge.

At the time of *C. auris* isolation, we recorded the length of prior hospitalization (in a general ward or ICU), previous or current receipt of antifungals, administration of glucocorticosteroids and application of a central venous catheter (CVC), mechanical ventilation, and renal replacement therapy.

We compared patients’ clinical characteristics and treatment-related factors among patients who were colonized and those infected, in order to determine potential predictors of a *C. auris* infection.

### 2.3. Environmental Sampling

The Infectious Disease Control Committee of the hospital was informed whenever a new case of *C. auris* isolation was identified. Environmental screening was performed when the first cluster of three patients occurred (during August–September 2021). This screening focused on the sampling of high-touch surfaces, such as beds and side tables, and also yielded this pathogen.

### 2.4. Identification of Isolates and Antifungal Susceptibility Testing

Yeasts from various clinical samples were identified and antifungal susceptibility testing was performed by the VITEK 2 Compact 15 automated system (Biomerieux, Marcy l’ Etoile, France) on a routine basis at the hospital clinical laboratory. Yeasts were grown on Sabouraud Dextrose agar at 35 °C and 42 °C. Additionally, CHROMagarTM Candida Plus agar was used and pale cream colonies with a distinctive blue halo, suspected as *C. auris*, were sent to the Department of Microbiology, Medical School, National and Kapodistrian University of Athens, Greece for further analysis. Identification of isolates was confirmed by MALDI-TOF spectrometry, which is one of the most efficient diagnostic techniques for accurate identification of *C. auris*, using the Microflex LT (Bruker Daltonics, Bremen, Germany) platform.

Susceptibility to antifungal agents was evaluated by the EUCAST standardized broth microdilution method [30]. Since there are no established breakpoints, interpretation of minimum inhibitory concentration (MIC) values was based on CDC proposed tentative breakpoints [26]. Therefore, the information below should be considered as a general guide and not as definitive breakpoints for resistance.

### 2.5. Statistical Analysis

Continuous variables were expressed as mean ± standard deviation (SD) or median (interquartile range). Continuous variables were compared with Student’s t test (for normally distributed variables) or the Mann–Whitney U test (for non-normally distributed variables). Categorical variables were evaluated with the χ^2^ or Fisher exact test. All tests were 2-tailed, and *p* < 0.05 was considered to indicate statistical significance.

## 3. Results

### 3.1. Patients’ Characteristics—Host Risk Factors (Table 1)

A total of 591 patients were admitted to the ICU and screened for *C. auris* between 10 November 2020 and 31 December 2023. During this study period, 32 patients were identified as being colonized (26 patients) or infected (6 patients) with *C. auris*. The mean age was 68 ± 14 years and males predominated (21 patients). Concerning disease severity, mean APACHE II score at ICU admission was 17.4 ± 6.9. All patients had been hospitalized in a general ward and/or in ICU before admission to our ICU [median time 13 (3–27) days]. All patients had at least one CVC, urinary catheter, arterial catheter, and nasogastric catheter and were on invasive mechanical ventilation. Four patients were on renal replacement therapy. All patients had received antibiotic treatment, whereas nine patients (28.1%) had recent history of antifungal therapy. Echinocandins had been administered in 5 (55.6%) patients, while azoles were prescribed in the rest of them (4; 44.4%). Duration of antifungal exposure was more than 8 days in all cases.

Initial diagnosis and reason for ICU admission were mainly medical (23 patients). The most common risk factors were cardiovascular disease (arterial hypertension, coronary artery disease, congestive heart failure) (15; 46.9%) and neuropsychiatric disorders (ischemic or hemorrhagic stroke, dementia, neurotic or psychotic disorders) (13;40.6%), followed by malignancy (11; 34.4%), diabetes (9; 28.1%), and chronic pulmonary disease (5; 15.6%). Two patients suffered from chronic renal failure and a total of five (15.6%) patients were immuno-compromised (on corticosteroids or chemotherapy treatment, or hematologic malignancy). COVID-19 infection and acute respiratory distress syndrome (ARDS) was the primary diagnosis for 11 (34.4%) patients.

After patients’ allocation in two groups according to *C. auris* colonization or infection, the sex distribution, mean age, and disease severity were similar between the groups. Regarding patient-related factors, no statistically significant differences in comorbidities were identified, despite the 2-fold higher incidence of diabetes and malignancies in the group of infected patients. Regarding treatment-related factors, the proportion of patients already receiving corticosteroids (34.6% vs. 83.3%, *p* = 0.064) or being on renal replacement treatment (7.7% vs. 33.3%, *p* = 0.15) were also higher in the group of infected patients. However, the observed differences did not reach statistical significance, probably due to the small size of groups.

### 3.2. Timeline of Cases

**Figure 1:**
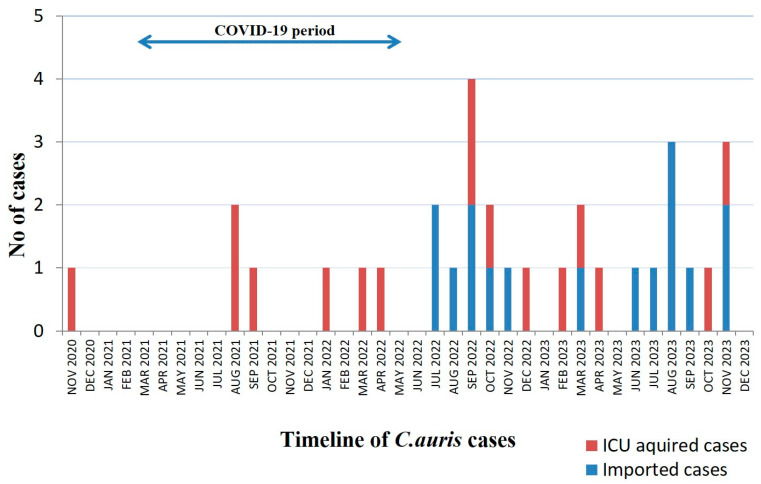
Timeline of 32 *C. auris* cases in ICU. The blue arrow denotes the period that the ICU has been functioning as a COVID-19 ICU.

Median time elapsed from ICU admission to first positive culture was 7 (1–21) days, whereas the corresponding time from hospital admission was 26 (19–49) days. Further analysis of the timeline of *C. auris* isolations revealed that half of cases occurred within 24 h after ICU admission, while the rest of them occurred after the first week of hospitalization in the ICU (Table 1). Out of the 16 imported *C. auris* cases, eight patients had been transferred from another ICU. The timeline of the 32 *C. auris* cases is presented in Figure 1.

### 3.3. Microbiological Data and Antifungal Susceptibility Testing

A total of 48 positive samples were recovered and one for each patient was analyzed when the *C. auris* strain was isolated simultaneously from colonized areas. Six patients had a growth of *C. auris* in the blood, whereas the rest of the isolates were allocated as follows: swab from axilla/groin 19 (39.58%); urine 9 (18.75%); tracheal aspirates 8 (16.67%); skin-soft tissue samples 3 (6.25%); central venous catheter (CVC) tip 3 (6.25%). Among our study population, 13 patients were colonized at two or more sites, out of which 6 patients *C. auris* emerged simultaneously at different sites. Out of six infections, *C. auris* colonization preceded in two cases (19 days and 39 days, respectively). None of the patients turned out negative until ICU discharge. All infected patients had suffered also from co-infections caused by multidrug-resistant bacteria (*Acinetobacter baumannii*, *Klebsiella pneumoniae*, *Enterococcus faecium*). In three cases, bacterial infection preceded the fungal infections, whereas in the rest, they followed.

Since all the strains shared almost identical susceptibilities. Based on tentative CDC MIC breakpoints for fluconazole (≥32 μg/mL), amphotericin B (≥2 μg/mL), micafungin (<4 μg/mL), anidulafungin (<4 μg/mL) and caspofungin (<2 μg/mL), all isolates were resistant to fluconazole (MICs > 32), and susceptible to echinocandines and amphotericin B (Table 2).

### 3.4. Therapeutic Strategies and Outcome Analysis

Out of a total of 32 patients, 17 did not receive any antifungal treatment after *C. auris* isolation. Out of 18 patients who were treated with antifungals, 9 received micafungin, 6 received caspofungin, 1 received anidulafungin, while 2 patients received combination therapy (anidulafungin or caspofungin plus amphotericin B).

Regarding source control, in the six cases of candidemia, the CVC was removed within 48 h of positive blood culture. In three cases where *C. auris* was isolated from skin/soft tissue samples, wound debridement was performed after 48 h of positive culture.

The all-cause mortality during ICU stay was 43.75%, whereas at 28 days after ICU discharge, this was 65.6%. All patients discharged alive from the ICU were transferred to general wards or ICUs within our other hospitals, according to the department where each patient had been initially admitted. Median ICU length of stay was 24 (13–45) days and was significantly longer for infected patients.

The crude mortality for the six patients with *C. auris* infection was 50%. However, only for one patient with bloodstream infection, death could be attributed to *C. auris*, whereas in the rest of cases, death was associated with multidrug-resistant bacteria. The mean time between the emergence of infection and ICU discharge was 36 ± 16 days and was also significantly longer when compared to colonized patients.

## 4. Discussion

In the present study, we reported an ongoing outbreak of *C. auris* colonization and infection in a Greek ICU. The introduction of *C. auris* in our ICU occurred before ICU’s operation as ICU-COVID -19 and represented a single case, since no other contemporary patients, nor the environmental samples, revealed *C. auris*. The next three cases occurred 10 months later, during the fourth wave of COVID-19 in Greece, when the ICU was operated as a COVID-19 ICU and clustered within a 25-day period. After a 4-month period, a new case of *C. auris* appeared and, thence, strains were isolated almost every month. After the introduction of *C. auris* in the healthcare setting in Greece in 2019, a nationwide outburst was observed. The National Public Health Organization (NPHO) of Greece recorded 429 cases during a 3-year period (November 2019 till December 2022) [31]. These isolates involved 45 public and private hospitals in Greece; 27 of them (60%) belonged to the district of Attiki. The majority of cases concerned colonization (314; 73.2%) and the crude mortality of affected patients was 28.4%. These data, however, might underestimate *C. auris* dissemination, given that reporting *C. auris* cases to the NHPO is still not mandatory. In a previous work [32], we have reported for the first time a series of five cases with *C. auris* that were clonally related, belonged to clade I (clustered with South-Asian strains).

One of the major problems regarding *C. auris* is its misidentification with conventional methodologies with other yeasts (e.g., *C. haemulonii*, *C. famata*, *C. sake*, *C. catenulata*, *Rhodotorula glutinis*, or *Saccharomyces cerevisiae*) [33]. The gold standard for *C. auris* identification is either advanced molecular methods, such as PCR and DNA sequencing, or MALDI-TOF mass spectrometry (MS). This latter methodology detects especially ribosomal proteins from the *Candida* surface and compares them to proteins from other yeast species using a large database and identifies *C. auris* with excellent specificity and sensitivity in a few minutes [33,34]. Of note, the US Food and Drug Administration (FDA) approved the Bruker MALDI Biotyper system (20 April 2018) and the bioMérieux Vitek MS (21 December 2018) for *C. auris* identification [34]. In our study, all the isolated yeasts strains were further analyzed by the aforementioned Bruker system.

The recent evolution of *C auris* can be associated with the increase in global temperatures, but also with the pandemic. Indeed, a major driver of *C. auris* healthcare-associated dissemination in Greece was COVID-19. Outbreaks of *C. auris* have been reported in COVID-19 patients worldwide, resulting in colonization or infection rates as high as 50% [35,36,37,38]. During the COVID-19 pandemic, extended use of the underlayer protective equipment, double gloving, poor adherence to hand hygiene, lapses in cleaning and disinfection procedures, along with low nurse-to-patient ratio, and inadequately trained staff recruited to work in ICUs may have contributed to widespread transmission of *C. auris* [37]. *C. auris* can form dense biofilms with up to 30-fold higher cellular burden than *C. albicans* [39], which are highly resistant to desiccation, osmotic stress, and disinfectants like chlorhexidine and can contaminate the skin of patients and healthcare workers [39,40]. Indeed, new patients become colonized with *C. auris* after a 4 h contact time with a carrier, while invasive infections have been described in patients within 48 h of ICU admission [41,42]. In our ICU, *C. auris* patients are isolated in single-person rooms and strict contact precautions are followed. However, the assignation of dedicated healthcare staff is not feasible and the low nurse-to-patient ratio, especially during night shifts, might have contributed to the horizontal transmission of *C. auris*.

An alarming issue in our series is the observed gap of 4 and 3 months between the discharge of a *C. auris* carrier and the subsequent emergence of a new one, during the time course of cases. Persistence of *C. auris* in the patient environment, along with undetected carriage by colonized patients or healthcare workers, might explain this time gap. *C. auris* biofilms can persist in viable colonies for ≥2 weeks and as viable nonculturable cells for ≥ 4 weeks [13]. In a recent observational multicenter study, positivity rates for colonization were 56% and 27% for groin and axilla, respectively [43]. Colonization of nares, groin, axilla, skin, urinary tract, vagina, and rectum with *C. auris* can last from 1 month to 3 years, and perhaps indefinitely [13,16,44]. Patients have been found to be colonized for several months after active infection has been resolved or may have intermittent negative results followed by a positive one. However, it has not been established yet if this timeframe differs between clinical cases and screening cases.

Through the present study, we investigated various host- and treatment-related factors, in order to identify potential risk factors that could predetermine *C. auris* infection. Similarly to previous workers [43,45], the most frequent comorbidities/risk factors in our case series were cardiovascular and neuropsychiatric disorders, malignancy, diabetes, COVID-19 infection, invasive mechanical ventilation, the presence of a CVC and urinary catheter, and a recent history of antibiotic and antifungal agents and corticosteroids. However, chronic comorbidities did not prove to affect patients’ colonization or infection status. Despite the fact that infected patients showed almost 2.5-fold higher rates of corticosteroid administration and 4.5-fold higher rates of renal replacement therapy compared with colonized patients, these differences did not reach statistical significance, probably due to the small size sample.

Nearly 10% of *C. auris* colonized patients develop invasive infections, particularly those with mechanical ventilation and placement of invasive devices in ICU settings [42,46,47]. Other investigators reported higher prevalence of candidemia (17%) among colonized patients, with an estimated cumulative incidence up to over 25% with increasing length of stay in critically ill patients [41,48,49]. In our case series, only two patients among the 28 already colonized patients develop *C. auris* bloodstream infection. However, the considerable time that elapsed before these patients became infected indicates this cumulative risk with extended hospitalization, especially in ICU setting.

*C. auris* has been implicated in a variety of infections, such as urinary tract infections, otitis, surgical wound infections, peritonitis, skin and bone infections, myocarditis, meningitis, and bloodstream infections [50,51]. However, most of the reported cases referred to bloodstream infections, as in our study.

Antifungal treatment management of *C. auris* infections is similar to other *Candida* species infections. Nevertheless, *C. auris* high-level resistance to antifungals is a major obstacle to successful management of the corresponding infections. Multidrug and pan drug resistance are seen in 41% and 3–4% of the strains [11,26,27]. Moreover, regional and clade-specific resistance variations have been reported. For example, clade II isolates show the highest fluconazole sensitivity rates of up to 86% [6]. On the contrary, clade I isolates, as in our case series, show the highest overall resistance with 97% to fluconazole, 54% to amphotericin B, and 49% presenting multidrug resistance [6]. Amphotericin B resistance has been observed in clades I and IV, with resistance rates as high as 50% [6,12]. *C. auris* employs multiple resistance mechanisms, such as drug target mutation or overexpression and biofilm formation. Both resistant and sensitive strains can coexist in the same population and genetically related isolates may convey different resistance alleles. Considering these clade-specific resistance variations, it is assumable that its high-level antifungal resistance is more likely to be an acquired trait rather than an intrinsic property [6,12,52]. Based on the aforementioned data, echinocandins are recommended as initial therapy for treatment of *C. auris* infections [26]. Similarly to recommendations for other *Candida* species, treatment of *C. auris* isolated from non-invasive sites (such as respiratory tract, urine, and skin colonization) is not encouraged when there is no evidence of infection [26]. The duration of antifungal treatment is also similar to those prescribed for infections caused by other *Candida* species and depends on clinical cure and source control, along with microbiological clearance [43]. In our study, infected patients received antifungal treatment for a median time of 27 days, along with source removal, such as CVC removal and wound debridement. Adequate and prompt source control is an essential intervention to improve treatment success, to reduce antifungal duration and possibly to prevent induction or selection of resistance. Indeed, there have been alarming reports of emerging resistance to echinocandins after a first treatment course with these antifungals, especially in cases of catheter-related infections [49,53,54]. As resistance can develop while patients are receiving therapy, repeat susceptibility testing is recommended to evaluate the need for an alternative agent [55].

Two of our infected patients, due to clinical deterioration, received combination therapy of an echinocandin (caspofungin or anidulafungin) with amphotericin B and a favorable outcome was achieved for one of them. Combinations of antifungals have been tested mostly in vitro. Synergism was noted for echinocandins and azoles combinations, especially for anidulafungin or micafungin with isavuconazole [56]. Jaggavarapu et al. demonstrated synergy between amphotericin B and micafungin in 8 among 10 tested strains [57]. Despite the limitations of in vitro synergy models, the activity of antifungal combinations may not be only species-specific but also strain-specific [56].

The crude ICU mortality of our case series was 43.75% and was similar for patients colonized (42.3%) and infected (50%), suggesting that *C. auris* may not have been directly implicated in an unfavorable outcome. Indeed, almost all infected patients died due to infection caused by multidrug-resistant bacteria. Biran et al. have also reported no differences in mortality among colonized and infected patients [45]. Crude mortality rates of 0 to 72% have been reported for *C. auris* infections, indicating heterogeneity of patients and studies [58]. In a meta-analysis, Chen et al. [59], reported that the pooled crude mortality of *C. auris* infection was 39%, with an overall mortality of bloodstream infections of 45%. Comparing this mortality with that of candidemia in Europe (38%) and that of multidrug-resistant *p. aeruginosa* (44.6%) and carbapenem-resistant *K. pneumoniae* (54.3%), they concluded that the mortality of *C. auris* candidemia was similar to other candidemias and some drug-resistant Gram-negative bacteremias. Since crude mortality might reflect the multiple acute and chronic comorbidities of critically ill patients, well-designed case–control studies should be carried out to estimate attributable mortality of *C. auris* more accurately.

Nevertheless, *C. auris* infections are associated with increased length of hospital stay and, in this way, may indirectly affect mortality [60]. Indeed, in our case series, a significant difference regarding the time elapsed from *C. auris* isolation to ICU discharge was observed among colonized and infected patients. Consequently, infection control policies are of paramount significance in order to contain the spreading of *C. auris* in the healthcare environment. Identifying patients or healthcare workers colonized with *C. auris* is the first step in impeding fungus transmission. High-risk patients and close healthcare contacts of patients with *C. auris* should be considered for screening for *C. auris* colonization. Patients on contact precautions should be either isolated in a single room or in a cohort with other *C. auris* patients and dedicated staff should be assigned. Strict contact precautions should include rigorous hand hygiene with alcohol or chlorhexidine rubs, personal protective equipment for healthcare staff, and dedicated medical equipment or single-use items. It is crucial to decontaminate regularly the high-touch areas along with the terminal cleaning and disinfection of patient environment after discharge [61,62] with high-strength (>1000 ppm) chlorine disinfectants or hydrogen peroxide with silver nitrate [26,40]

Additionally, there is no cumulative evidence regarding the effectiveness of protocols for the decolonization of patients with *C. auris* [26]. The antimicrobial stewardship team could check for the unnecessary management of cases. The cooperation of the antimicrobial stewardship team with the infection control team is essential in order to limit *C. auris* transmission [63]. Finally, information on *C. auris* infection or colonization should be communicated whenever patients are transferred to lower levels of care, in order to ensure all appropriate infection control measures are continued.

*C. auris* is capable of colonizing up to 90% of asymptomatic humans, while adopting commensal lifestyle or the pathogen (pathobiont). The detection of its passage into pathological mode is very difficult and depends on various host factors, along with *C. auris* genetic potential and its relationships with neighboring microbes. Also, *C.auris* undergoes micro-diversification on its host on the timescale of months [55]. Further genomic and clinical studies could elucidate the unanswered questions and promote therapeutic modalities that will keep *C. auris* out of its pathogenic state.

## 5. Limitations—Conclusions

The present study has some limitations that should be considered. The observational and retrospective nature of the study brings about an intrinsic limitation. Since it is a single-center study, susceptibility patterns and management practices might have influenced our conclusions, and the results may differ according to the settings of different ICUs. The sample size to analyze the anti-fungal MIC distribution of *C. auris* isolates is not large. The low number of infected patients may have underestimated the causal role of certain risk factors and hampered further mortality analysis. Nevertheless, this study represents a real-life clinical experience that provides useful data regarding this emerging yeast. It demonstrates several clinical characteristics that render critically ill patients vulnerable for *C. auris* acquisition. It appoints the high resistance of *C. auris* to fluconazole and the excellent sensitivity of echinocandins, leading to administration of these antifungals as the first choice. The crude mortality of our study population was similar to other candidemias and some Gram-negative drug-resistant bacteria previously reported, and the attributable mortality was even lower. *C. auris* may not be so scary, however it should not be underestimated. Rapid identification of *C. auris*, continued surveillance, and infection control practices, such as screening of close contacts, contact precautions, and proper terminal cleaning, are the most important elements for controlling successfully its spread in the hospital setting. Each patient’s reaction to *C. auris* may differ according to host inner immunity, different *C. auris* strains and local environment. Implementation of individualized and prompt therapy is necessary in order to avoid antifungal consumption and potential micro-diversification of *C. auris*.

## Figures and Tables

**Table 1:** Risk factors and characteristics of colonized or infected patients ^a^.

Risk Factor	All Patients (n = 32)	Colonized Patients (n = 26)	Infected Patients (n = 6)	*p*-Value
Demographics				
Gender	21 males (65.6) 11 females (34.4)	17 males (65.4) 9 females (34.6)	4 males (66.7) 2 females (33.3)	0.724
Age (years) (mean ± S.D.)	68 ± 14	69 ± 13	61 ± 16	0.189
APACHE II score (mean ± S.D.) ^b^	17.4 ± 6.9	17.4 ± 6.3	17.3 ± 9.5	0.987
Initial diagnosis ^b^	23 medical (71.9) 9 surgical (28.1)	18 medical (69.2) 8 surgical (30.8)	5 medical (83.3) 1 surgical (16.7)	0.648
Prior LOS in hospital (days) [median (IQR)]	13 (3–27)	14 (5–46)	6 (3–10)	0.154
Comorbidities				
COVID-19	11 (34.4)	9 (34.6)	2 (33.3)	0.679
Cardiovascular disease	15 (46.9)	14 (53.8)	1 (16.7)	0.178
Chronic pulmonary disease	5 (15.6)	4 (15.4)	1 (16.7)	0.585
Neuropsychiatric disorders	13 (40.6)	13 (50)	0	0.059
Diabetes	9 (28.1)	6 (23.1)	3 (50)	0.314
Chronic renal failure	2 (6.2)	2 (7.7)	0	0.815
Malignancy	11 (34.4)	7 (26.9)	4 (66.7)	0.148
Immunosuppression ^c^	5 (15.6)	4 (15.4)	1 (16.7)	0.585
On isolation day—treatment-related factors				
ICU day	7 (1–21)	2 (1–13)	25 (19–54)	0.005
Corticosteroids	14 (43.75)	9 (34.6)	5 (83.3)	0.064
Renal replacement therapy	4 (12.5)	2 (7.7)	2 (33.3)	0.15
Prior antifungal administration	9 (28.1)	8 (30.8)	1 (16.7)	0.648
Outcome data				
ICU LOS (days)	24 (13–45)	20 (12–37)	52 (44–100)	0.003
Time elapsed between 1st isolation and ICU discharge (days) (mean ± S.D.)	18 ± 14	14 ± 10	36 ± 16	<0.001
ICU survival	18 (56.2)	15 (57.7)	3 (50)	1.0
Survival on the 28th day after ICU discharge	11 (34.4)	9 (34.6)	2 (33.3)	1.0

S.D., standard deviation; APACHE, Acute Physiology and Chronic Health Evaluation; LOS, length of stay; IQR, interquartile range; ICU, intensive care unit; ^a^ Data are no. (%) of patients unless otherwise stated. ^b^ At ICU admission. ^c^ Neutropenia (neutrophil count < 1000/mm^3^), immunosuppressant medication (including corticosteroids), splenectomy.

**Table 2:** Minimum inhibitory concentration values (MICs) of *Candida auris* strains isolated from the samples of 32 patients ^a^.

Number of Patient ^c^	Gender	Type of Specimen Positive for *Candida auris*	APHB ^b^	ANF	MIF	CAS	ISA	POS	VOR	ITZ	FCA
1	Female	Hip decubitus ulcer	0.5	0.12	0.5	0.12	0.03	0.25	0.25	0.12	128
		Urine	^-^	^--^	^-^	^-^	^-^	^-^	^-^	^-^	^-^
2	Female	Urine (ureterostomy)	1	0.5	0.5	1	0.03	0.5	8	1	256
3	Female	Urine	1	0.12	0.12	0.12	0.06	0.25	8	0.5	>256
4	Male	Blood	0.5	0.12	0.12	0.5	0.03	0.25	0.5	0.25	256
5	Female	Tracheal aspirates	1	0.25	0.25	0.12	0.06	0.12	0.12	0.06	256
6	Female	Blood	1	0.5	0.25	0.5	0.03	0.5	8	0.5	>256
		Urine	1	0.5	0.25	0.5	0.03	0.5	8	0.5	>256
		Tracheal aspirates	^-^	^--^	^-^	^-^	^-^	^-^	^-^	^-^	^-^
7	Male	Urine	0.5	0.25	0.25	0.5	<0.008	0.03	0.05	0.12	128
8	Male	Swab from axilla/groin	1	0.06	0.06	0.12	0.06	0.06	0.25	0.12	128
		Central venous catheter	11	0.06	0.06	0.12	0.06	0.06	0.25	0.12	256
9	Female	Swab from axilla/groin	1	0.5	0.5	0.25	0.03	0.12	0.5	0.12	32
10	Male	Swab from axilla/groin	0.5	0.5	0.5	0.5	0.12	0.25	8	0.5	256
		Urine	1	0.5	0.5	0.5	0.12	.25	8	0.5	>256
11	Female	Swab from axilla/groin	1	0.12	0.12	0.5	0.03	0.06	0.5	0.12	128
12	Male	Hip decubitus ulcer	1	0.25	0.25	0.25	0.06	0.25	0.25	0.25	32
13	Male	Swab from axilla/groin	1	0.25	0.25	0.5	0.06	0.015	2	0.25	32
14	Female	Blood	1	0.5	0.5	1	0.06	0.25	8	0.5	>256
		Central venous catheter	1	0.5	0.5	1	0.06	0.25	8	0.5	>256
15	Female	Swab from axilla/groin	1	0.5	0.5	1	0.03	0.25	8	0.5	>256
16	Male	Swab from axilla/groin	1	0.5	0.5	1	0.12	0.5	8	0.5	>256
17	Male	Swab from axilla/groin	0.5	0.5	0.5	1	0.12	0.5	8	0.5	>256
		Tracheal aspirates	^-^	^--^	^-^	^-^	^-^	^-^	^-^	^-^	^-^
18	Male	Tracheal aspirates	1	0.5	0.25	0.5	0.06	0.06	1	0.25	>256
19	Female	Swab from axilla/groin	1	0.25	0.25	0.25	0.015	0.12	2	0.25	>256
		Tracheal aspirates	^-^	^--^	^-^	^-^	^-^	^-^	^-^	^-^	^-^
20	Male	Blood	1	0.12	0.12	0.25	0.03	0.25	2	0.25	>256
		Swab from axilla/groin	^-^	^--^	^-^	^-^	^-^	^-^	^-^	^-^	^-^
21	Male	Swab from axilla/groin	1	0.12	0.12	0.25	0.03	0.06	1	0.12	128
22	Male	Blood	1	0.12	0.12	0.06	0.03	0.03	0.5	0.12	128
23	Male	Swab from axilla/groin	1	0.12	0.12	0.25	0.06	0.06	0.5	0.25	128
		Leg decubitus ulcer	^-^	^--^	^-^	^-^	^-^	^-^	^-^	^-^	^-^
24	Male	Swab from axilla/groin	0.5	0.12	0.03	0.12	<0.008	<0.008	0.12	0.03	64
25	Male	Swab from axilla/groin	1	0.12	0.012	0.5	0.06	0.06	0.5	0.12	>256
26	Male	Swab from axilla/groin	1	0.12	0.03	0.5	0.06	0.06	0.5	0.25	128
27	Female	Swab from axilla/groin	0.5	0.12	0.12	0.5	0.015	0.03	0.25	0.12	128
		Urine	^-^	^--^	^-^	^-^	^-^	^-^	^-^	^-^	^-^
28	Male	Swab from axilla/groin	1	0.5	0.12	0.5	0.03	0.06	0.5	0.12	64
		Tracheal aspirates	^-^	^--^	^-^	^-^	^-^	^-^	^-^	^-^	^-^
29	Male	Blood	1	0.12	0.03	0.5	0.015	0.03	0.5	0.12	128
		Central venous catheter	1	0.12	0.03	0.5	0.015	0.03	0.5	0.12	128
30	Male	Swab from axilla/groin	0.5	0.12	0.12	0.25	0.03	0.03	0.5	0.25	128
		Urine	^-^	^--^	^-^	^-^	^-^	^-^	^-^	^-^	^-^
		Tracheal aspirates	^-^	^--^	^-^	^-^	^-^	^-^	^-^	^-^	^-^
31	Male	Urine	0.5	0.06	0.12	0.12	0.03	0.06	0.25	0.25	256
32	Male	Swab from axilla/groin	^-^	^--^	^-^	^-^	^-^	^-^	^-^	^-^	^-^
		Tracheal aspirates	1	0.12	0.12	0.25	0.015	0.03	1	0.5	64

^a^ No MIC values in simultaneously isolated strains from colonized sites (patients n. 1, 6, 17, 19, 20, 23, 27, 28, 30, 32). ^b^ APHB: amphotericin B; ANF: anidulafungin; MIF: micafungin; CAS: caspofungin; ISA; isavuconazole; POS: posaconazole; VOR: voriconazole; ITZ: itraconazole; FCA: fluconazole. ^c^ Patients n. 4, 6, 14, 20, 22, 29 had growth of *C. auris* in the blood culture; patients n. 1, 6, 8, 10, 14, 17, 19, 20, 23, 27, 28, 30, 32 were colonized at two or more sites; patients n. 1, 10, 17, 23, 30, 32 were colonized simultaneously at different sites; in patients n.6 and n.20, *C. auris* colonization preceded 19 and 39 days, respectively.

## Data Availability

The data presented in this study are available on request from the corresponding authors due to ethical reasons
